# Open-Air Sanatorium and Graduated Labour

**Published:** 1910-07-09

**Authors:** C. Muther

**Affiliations:** Mendip Hills Sanatorium, Wells.


### OPEN-AIR SANATORIUM AND GRADUATED LABOUR.

Sir,—In the excellent article on "Sanatorium Management" that appeared in your last issue, the writer unwittingly conveys one or two wrong impressions on graduated labour. He says, in common with many others, that the system of graduated labour was first introduced by the medical men in connection with Frimley Sanatorium. I have been carrying on the sanatorium treatment for the last eleven or twelve years, and graduated manual exercises, together with breathing and singing exercises, have formed a part of the daily programme of our patients at least during the last six years—in fact, before Frimley Sanatorium came into being. Ever since the introduction of manual exercises we have noticed a marked improvement in our results, so much so that I would strongly advise that they should be given a fair trial in every sanatorium. My long experience enables me to say that a consumptive patient should work out his salvation by work—of course under medical supervision. We only employ three grades of labour, which are as follows :—

First grade.—Weeding in the garden, hoeing, raking and gathering leaves, edging grass, planting, pruning, etc.

Second grade.—Chopping logs, making paths in the woods, spade work, digging broken ground, etc.

Third grade.—Sawing, carpentry, painting, digging unbroken ground, wheelbarrow work, etc. (*vide* p. 98 in my book on Pulmonary Tuberculosis).

For private patients these three grades are quite sufficient to obtain the full physiological effect of work.

Yours truly,

Mendip Hills Sanatorium, Wells. C. Muther.

